# Frontal cerebral oxygenation asymmetry: intersubject variability and dependence on systemic physiology, season, and time of day

**DOI:** 10.1117/1.NPh.7.2.025006

**Published:** 2020-06-23

**Authors:** Hamoon Zohdi, Felix Scholkmann, Ursula Wolf

**Affiliations:** a University of Bern, Institute of Complementary and Integrative Medicine, Bern, Switzerland; b University of Zurich, University Hospital Zurich, Biomedical Optics Research Laboratory, Department of Neonatology, Zurich, Switzerland

**Keywords:** functional near-infrared spectroscopy, prefrontal cortex, right–left asymmetry, tissue oxygenation, systemic physiology

## Abstract

**Significance:** Our study reveals that frontal cerebral oxygenation asymmetry (FCOA), i.e. a difference in the oxygenation between the right and left prefrontal cortex (PFC), is a real phenomenon in healthy human subjects at rest.

**Aim:** To investigate FCOA, we performed a study with 134 healthy right-handed subjects with the systemic physiology augmented functional near infrared spectroscopy (SPA-fNIRS) approach.

**Approach:** Subjects were measured 2 to 4 times on different days resulting in an unprecedented number of 518 single measurements of the absolute values of tissue oxygen saturation (${\mathrm{StO}}_{2}$) and total hemoglobin concentration ([tHb]) of the right and left PFC. Measurements were performed with frequency-domain functional near-infrared spectroscopy. In addition, the cardiorespiratory parameters were measured simultaneously.

**Results:** We found that (i) subjects showed an FCOA (higher ${\mathrm{StO}}_{2}$ on the right PFC), but not for tHb; (ii) intrasubject variability was excellent for both ${\mathrm{StO}}_{2}$ and tHb, and fair for FCOA; (iii) ${\mathrm{StO}}_{2}$ correlated significantly with blood ${\mathrm{CO}}_{2}$ concentration, [tHb] with heart rate, respiration rate (RR), and the pulse–respiration quotient (PRQ), and FCOA with RR and PRQ; (iv) FCOA and ${\mathrm{StO}}_{2}$ were dependent on season and time of day, respectively; (v) FCOA was negatively correlated with the room temperature; and (vi) ${\mathrm{StO}}_{2}$ and tHb were not correlated with the subjects mood but with their chronotype, whereas FCOA was not dependent on the chronotype.

**Conclusion:** Our study demonstrates that FCOA is real, and it provides unique insights into this remarkable phenomenon.

## 1 Introduction

Our own preliminary measurements suggested that cerebral oxygenation differed between the right and left prefrontal cortex (PFC) in healthy human adults at rest.^1^ This phenomenon, which we named frontal cerebral oxygenation asymmetry (FCOA), is characterized by higher tissue oxygenation over the right PFC compared to the left.

Hemispheric specialization has been reported for a wide range of cerebral functions.^2^ For example, it is known that the regions in the left hemisphere are usually dominant for language and logical processing, whereas regions in the right hemisphere are specialized for spatial recognition and emotional control.^3^^,^^4^ Lateralization of function was first reported in the domain of language functions, which is widely accepted as a fundamental feature of neural organization, where it was revealed that the left hemisphere is dominant in language processing.^5^^,^^6^ Additionally, more than 90% of the population prefers the right hand for manual activities, with superior fine motor control and motor strength, which is controlled by the left hemisphere.^4^ Research has demonstrated that neural organization exists for the control motor actions, where each brain hemisphere contributes exclusive control mechanisms to the movement of each arm.^7^ Mutha et al.^7^ suggested that the left hemisphere provides predictive control mechanisms, whereas the right one contributes positional control mechanisms during movement of either arm. In addition to lateralization of language and motor control, face processing has also been shown to have laterality to neural activity and connectivity. It has been demonstrated that areas in the right hemisphere are more anatomically connected, more synchronized during rest, and more actively communicating with each other during face perception compared to the left hemisphere.^8^ Interestingly, numerous electroencephalography (EEG) studies have demonstrated a right–left asymmetry in brain activity during the resting state. Frontal EEG asymmetry (FEA) activity has been explained using the approach-withdrawal model suggesting that there are two different types of motivation.^9^^,^^10^ The approach motivation signifies the propensity to move toward the desired stimulus and is associated with a higher left frontal activity, whereas the withdrawal/avoidance motivation indicates a propensity to move or stay away from an undesired stimulus and is associated with higher right frontal activity.^11^^–^^13^ However, only a few studies on the asymmetry of brain tissue oxygenation and metabolism have been performed so far.^1^^,^^14^ This prompted us to investigate this fascinating phenomenon in many subjects using functional near-infrared spectroscopy (fNIRS).

From a methodological point of view, three main types of NIRS-based optical tissue spectroscopy techniques have been developed so far: continuous wave (CW-NIRS), frequency domain (FD-NIRS), and time domain (TD-NIRS). CW-NIRS can provide information on concentration changes of oxyhemoglobin ($\left[O_{2}\mathrm{Hb}\right]$) and deoxyhemoglobin ([HHb]) but cannot determine absolute baseline values. Therefore, it is appropriate for applications in cognitive neuroscience as absolute values are not crucial and functional activity is relatively assessed with respect to the baseline.^15^ FD-NIRS and TD-NIRS measure not only the light intensity as CW NIRS but also the time of flight of photons through tissue. Therefore, time resolved techniques such as TD-NIRS and FD-NIRS are able to provide absolute $\left[O_{2}\mathrm{Hb}\right]$, [HHb], and total hemoglobin ([tHb]) concentrations as well as absolute tissue oxygen saturation $\left[{\mathrm{StO}}_{2}=([O_{2}\mathrm{Hb}]/[\mathrm{tHb}])\times 100\right]$.^16^^,^^17^ This is relevant additional information, e.g., the [tHb] is strictly proportional to cerebral blood volume by the hematocrit. Thus these systems have widely been used in many diverse fields and applications including clinical monitoring, traumatic brain injury, anesthesiology, neonatology, and psychiatry.^18^ A comprehensive review on the history of fNIRS development, methodology, and imaging instrumentation has been published by Scholkmann et al.^19^ In this study, we performed optical neuroimaging using multidistance FD-NIRS. This approach is also able to reduce the sensitivity to extracerebral tissue. It is known that the oxygenation of the brain depends on its activity state, and the metabolic changes in the brain are interrelated with systemic parameters.^20^^–^^22^ Therefore, it is essential to employ the systemic physiology augmented (SPA) fNIRS approach, which additionally and simultaneously measures absolute values of cardiorespiratory parameters including the end-tidal carbon dioxide ($P_{\mathrm{ET}}{\mathrm{CO}}_{2}$), heart rate (HR), respiration rate (RR), and the pulse-respiration quotient (PRQ).

The main goal of this study was to investigate FCOA in a large number of healthy humans at rest to elucidate whether FCOA is a real and robust phenomenon. To facilitate a better understanding of this phenomenon, we employed SPA-fNIRS to assess whether FCOA depends on systemic physiological activity, absolute tissue oxygenation, or hemoglobin concentration. We also aimed to explore the effects of chronobiological and psychological variables on FCOA, as well as cerebral hemodynamics and oxygenation at the PFC during the resting state.

## 2 Subjects and Methods

### 2.1 Subjects

The study was carried out with 134 healthy subjects (85 female, 49 male, age 24.7±3.4 years, and range 20 to 46 years). The subjects were all right-handed, according to the Edinburgh Handedness Inventory.^23^ Subjects were nonsmoking and indicated neither current nor previous history of neurological and psychiatric disorders or alcohol and drug abuse. Subjects were asked to refrain from consuming caffeine and eating 2 h prior to the experiment. The study protocol was approved by the Ethics Committee of the Canton of Bern. Informed consent was obtained from all subjects before the measurements. Subjects were also informed of their right to discontinue participation at any time.

### 2.2 Experimental Protocol

The resting state data were taken from a set of studies with different stimuli. Each measurement began with a baseline phase lasting 8 min, during which the subjects sat upright in a comfortable chair in a dark room. For this study, we examined only the last 5 min of this baseline period. Each subject was measured on four different days but at the same time of day to prevent chronobiological artifacts. They were asked to keep their eyes open throughout the entire measurement and to move their head or body as little as possible during the measurement to avoid movement artifacts. Additionally, the subjects were asked to fill out two questionnaires before and after each measurement in order to assess their mood: the positive affect negative affect schedule (PANAS)^24^ and the self-assessment manikin test (SAM; five points scale).^25^ The PANAS and SAM questionnaires are used as tools to measure state influence. Trait influence evaluation was not performed in our study. Additionally, we determined the chronotype by the Horne and Östberg morningness-eveningness questionnaire.^26^ Measurements were performed between 7:00 am and 9:00 pm. The mean room temperature was 22.8°C±0.6°C.

### 2.3 Measurement Setup

The Imagent (ISS Inc., Champaign, Illinois, USA), a multichannel FD-NIRS system, which employs a multidistance approach, was used to determine absolute values of the $\left[O_{2}\mathrm{Hb}\right]$, [HHb], [tHb], and ${\mathrm{StO}}_{2}$ at a sampling rate of 2.5 Hz on the PFC. The Imagent’s light source consists of 16 laser diodes at 760 nm and 16 laser diodes at 830 nm. Four highly sensitive photomultiplier tubes serve as detectors. The sensors were placed bilaterally on the left and right prefrontal cortex (L-PFC and R-PFC) of subjects at position Fp1 and Fp2, according to the international 10 to 20 system.^27^ Each of the two ISS sensors had four light emitters and one light detector connected to an optical fiber delivering the light to the photomultiplier tube. The source–detector separations (d) were ∼2.0, 2.5, 3.5, and 4.0 cm with the sources and detectors arranged collinearly.

HR was measured by SOMNOtouch™ NIBP (SOMNOmedics GmbH, Randersacker, Germany) with a sampling rate of 4 Hz. This device calculated the HR from the ECG data by calculating the R−R intervals. RR and end-tidal carbon dioxide ($P_{\mathrm{ET}}{\mathrm{CO}}_{2}$) were measured noninvasively by a NONIN LifeSense (NONIN Medical, Plymouth, Minnesota, USA). Data were recorded at a sampling rate of 1 Hz. All data were recorded simultaneously.

### 2.4 Signal Processing and Statistical Analysis

One subject was excluded from data analysis due to the perceived discomfort of the fNIRS sensors. 126 subjects completed all four measurements; only for seven subjects, the number of experimental sessions was lower. Therefore, the entire data for the current analysis comprised 518 single measurements. All signal processing was performed in MATLAB (R2017a, MathWorks, Inc., Massachusetts, USA).

#### 2.4.1 Cerebral oxygenation and hemodynamics

Prior to analysis, data with extremely low (${\mathrm{StO}}_{2}<40\%$) or improper high values (${\mathrm{StO}}_{2}>100\%$) were removed by visual inspection. Movement artifacts in the ${\mathrm{StO}}_{2}$ and [tHb] signals were removed by the movement artifact reduction algorithm (MARA) based on moving standard deviation and piecewise-interpolation.^28^ For 86% of the signal time series, no processing with MARA was necessary. To remove high-frequency noise, signals were low pass filtered using a robust second-degree polynomial moving average (RLOESS) with a span of 2 min. This method assigns zero weight to data outside six mean absolute deviations (MAD). For each measurement, the 5-min median of the baseline phase was calculated for each cerebral parameter. Absolute values of ${\mathrm{StO}}_{2}$ and [tHb] from the L-PFC and R-PFC were averaged to obtain a single value for the whole PFC. Moreover, the laterality index—defined as the difference between the absolute values for the R-PFC and L-PFC—was determined and is indicated by a “Δ.” Finally, median values and the interquartile range (IQR) of ${\mathrm{StO}}_{2}$ and [tHb] were calculated for each individual subject.

#### 2.4.2 Cardiorespiratory parameters

All cardiorespiratory parameters, including HR, $P_{\mathrm{ET}}{\mathrm{CO}}_{2}$, and RR, were also denoised by the RLOESS method with a window length of 3, 1, and 2 min, respectively. Additionally, the PRQ (= HR/RR) was calculated to quantify the coupling between HR and RR. The 5-min median of the baseline phase was determined for all systemic physiology data. The IQR of parameters was calculated for each individual subject.

#### 2.4.3 Statistical analysis

Outliers (defined as exceeding three scaled MAD from the median) of each dataset were removed prior to the correlation analysis. The best nonlinear curve fitting (from many models including line, poly, cubic, degree 4 and 5 polynomial, piecewise linear function with 2 segments, and exponential) was estimated with R statistical software (R 3.5.2, Performance Analytics package, r-project.org) and OriginPro (version 2018b, OriginLab Corporation, Northampton, Massachusetts, USA) for each pair of parameters (8 parameters and 28 pairs) and a robust nonlinear regression was then calculated with MATLAB using the least absolute residuals method in order to avoid false-positive correlation detection. P-values were then obtained from goodness-of-fit results of each parameter pair. A false discovery rate (FDR) correction was subsequently applied to the p-values in order to correct for the multiple comparison situation. The bootstrapped evidence (BSE) test was conducted to find bootstrapped correlations between all parameters (28 pairs). This nonparametric method is an actual resampling procedure that takes the precision with which both the experimental ($H_{1}$) and null ($H_{0}$) hypothesis can be estimated into account.^29^ This test is also more robust compared to classical statistics by minimizing false positives while maintaining sensitivity. To investigate the dependence of cerebral parameters on seasonal changes, we applied the analysis of covariance (ANCOVA) by JASP (jasp-stats.org, version 0.9.2.0). ANCOVA is appropriate to test the main and interaction effects of categorical variables (covariates) on a continuous dependent variable. In this analysis, age and sex were selected as covariates, and a cerebral parameter and season were chosen as dependent and fixed factors, respectively. Since ANCOVA (Kruskal–Wallis nonparametric test; Dunn’s *post hoc* comparisons; Holm correction) showed that the covariates (sex and age) have interaction effects on most cerebral variables, the effect of seasonal changes, time of day, and temperature on cerebral parameters were investigated separately for both female and male groups. For this analysis, eight subjects aged over 30 years were excluded from these evaluations in order to have a sample in a small age range (20 to 30 years of age). Finally, Cosinor analysis [Eq. (1)] and the sum of 2 cosine functions [Eq. (2)] were applied in order to find the best chronobiological fit model of the cerebral parameters:

$$f(t)=M+A\;\cos[\frac{2\pi (t+\varphi )}{24}],$$ (1)

where f(t) denotes the value of the function at time t (e.g., a cerebral parameter), M is the midline estimating statistic of rhythm, A is the amplitude, t is measured in hours, and φ is the acrophase:

$$f(t)=\text{base}+A_{1}\;\cos[\frac{2\pi (t+\varphi )}{24}]+A_{2}\;\cos[\frac{2\pi (t+\varphi )}{12}].\;$$ (2)

where f(t) represents the value of the function at time t, base is the cerebral parameter baseline value, $A_{1}$ and $A_{2}$ are the amplitudes of the cosine functions, and φ is the acrophase.

#### 2.4.4 Reliability analysis

The intrasubject variability of all data, including cerebral and cardiorespiratory parameters were analyzed by the intraclass correlation coefficient (ICC) using the R statistical software (R 3.5.2, ICC package, r-project.org). ICC is a more desirable measure of reliability, reflecting both degrees of correlation and agreement between measurements. According to Fleiss,^30^ ICC values <0.4, between 0.4 and 0.6, in the range of 0.6 and 0.75, and >0.75 are indicative of poor, moderate, good, and excellent reliability, respectively.

## 3 Results

### 3.1 Cerebral Oxygenation and Perfusion: Higher StO_2_ of Right PFC

**Fig. 1:**
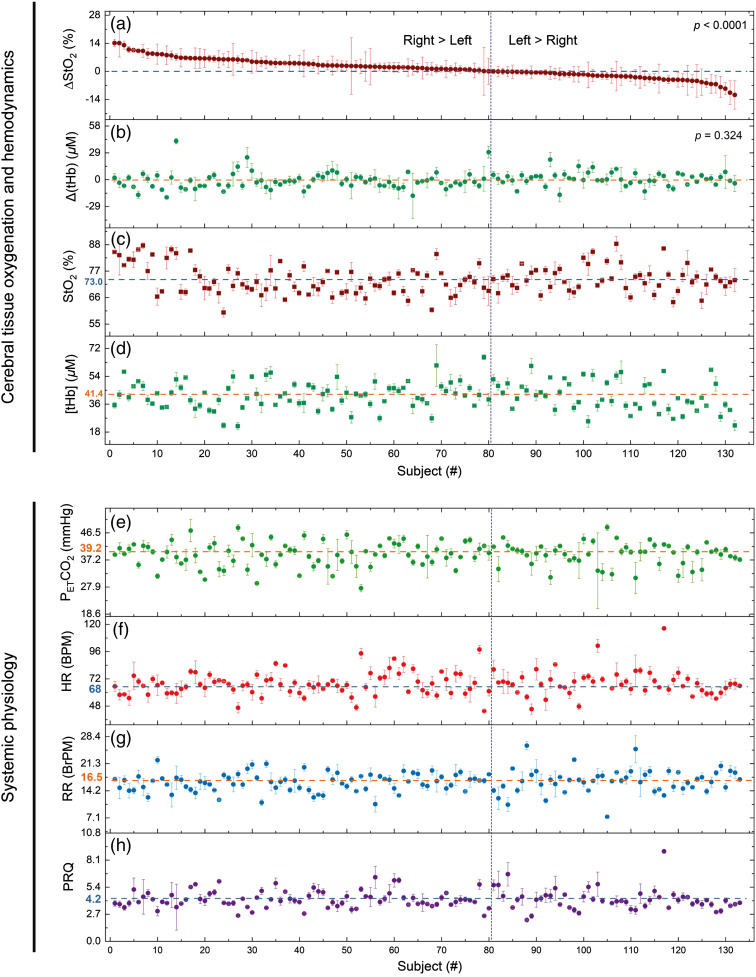
(a) FCOA of ${\mathrm{StO}}_{2}$ at the PFC sorted in descending order on the individual subjects. (b) Asymmetry of [tHb] and absolute values of (c) ${\mathrm{StO}}_{2}$, (d) [tHb], (e) $P_{\mathrm{ET}}{\mathrm{CO}}_{2}$, (f) HR, (g) RR, and (h) PRQ displayed according to the $\Delta {\mathrm{StO}}_{2}$ sorting, at the PFC for individual subjects during resting state. The median and the IQR are shown for each subject.

**Fig. 2:**
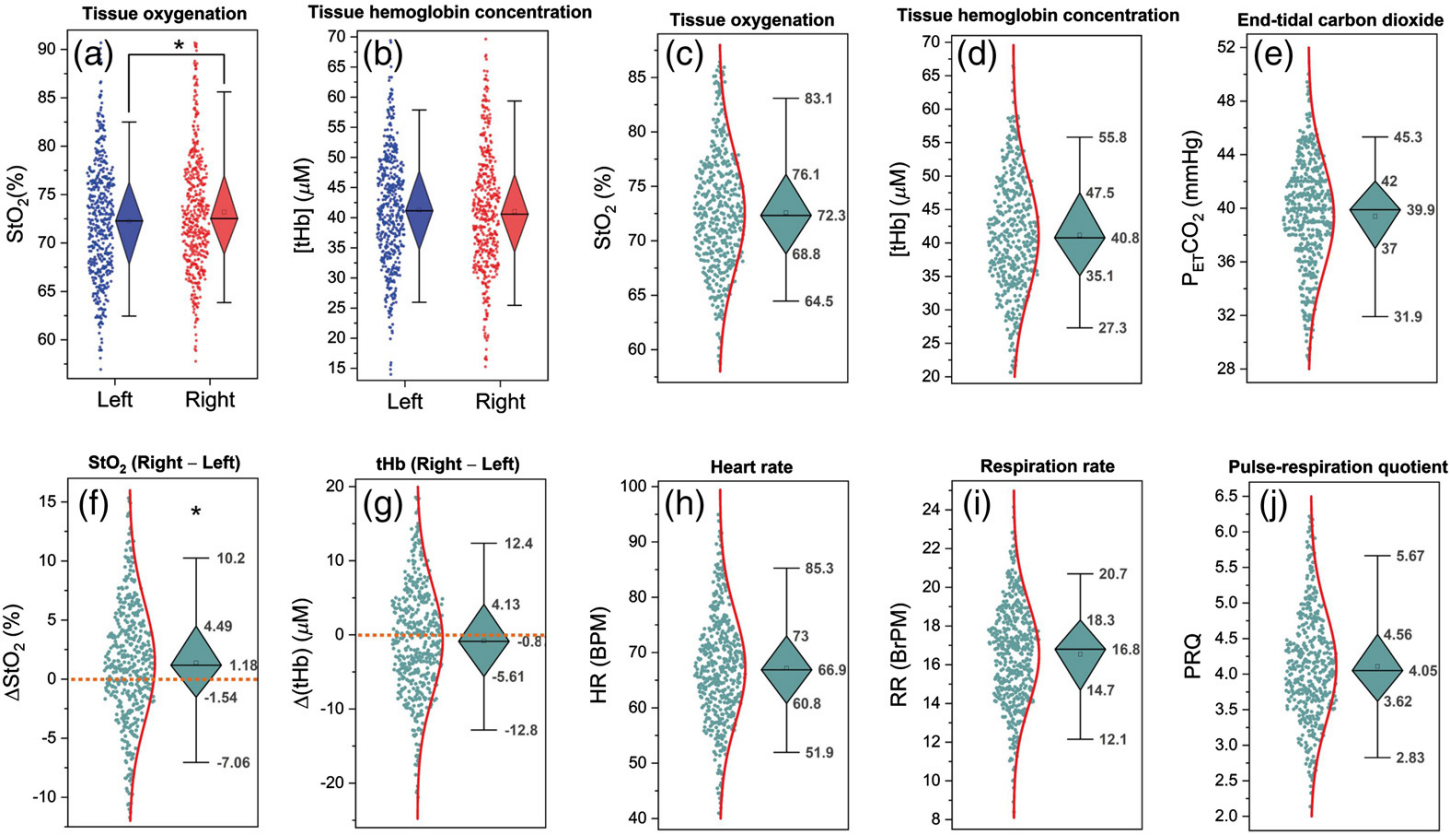
Diamond box plots showing distributions of absolute (a) ${\mathrm{StO}}_{2}$ and (b) [tHb] values at the R-PFC and L-PFC, (c) ${\mathrm{StO}}_{2}$, (d) [tHb], (e) $P_{\mathrm{ET}}{\mathrm{CO}}_{2}$, (f) $\Delta {\mathrm{StO}}_{2}$, (g) ΔtHb, (h) HR, (i) RR, and (j) PRQ values in resting state. The diamond spans the first quartile to the third quartile (IQR). A segment inside the diamond shows the median and whiskers above and below the box plots represent the 95% prediction interval. The asterisks indicate the level of high significance between absolute ${\mathrm{StO}}_{2}$ values of the R-PFC and L-PFC (*p<0.001, Wilcoxon signed-rank test). Outliers are not displayed.

For ${\mathrm{StO}}_{2}$, we found right-dominant activity ($\Delta {\mathrm{StO}}_{2}>0$), i.e., a highly significant (p<0.0001) FCOA was detected in the resting state [Fig. 1(a)]. No significant (p=0.324) asymmetry was found for [tHb] [Fig. 1(b)]. The intersubject mean value representing the normal value of ${\mathrm{StO}}_{2}$ and [tHb] was (mean±SD) 73.0%±5.9% and 41.4±9.3  μM, respectively [Figs. 1(c) and 1(d)]. For the right and left PFC the normal values of ${\mathrm{StO}}_{2}$ were 73.7%±6.9% (right) and 72.3%±6.1% (left), and of [tHb] 41.4±10.8 (right) and 41.4±10.1 (left) [Figs. 2(a) and 2(b)]. The absolute values of ${\mathrm{StO}}_{2}$ and [tHb] during the resting state are shown in Figs. 2(c) and 2(d). All data were normally distributed. From this point on, the outliers were removed and not considered for subsequent evaluations. After removal of the outliers, FCOA remained highly significant (p<0.0001) [nonsignificant for [tHb] (p=0.218)].

### 3.2 Cardiorespiratory Activity

Figures 1(e)–1(h) and 2(e), 2(h)–2(j) show the absolute values of $P_{\mathrm{ET}}{\mathrm{CO}}_{2}$, HR, RR, and PRQ for the individual subjects during resting state. On the group level, mean absolute and SD values of cardiorespiratory parameters were as follows (data were normally distributed): $P_{\mathrm{ET}}{\mathrm{CO}}_{2}$: 39.2±4.4  mmHg, HR: 68±11 beats/min (BPM), RR: 16.5±2.9 breaths/min (BrPM), and PRQ: 4.2±0.9. These values were all in the normal range for healthy adults at rest.

### 3.3 Relationships with Systemic Physiology

**Fig. 3:**
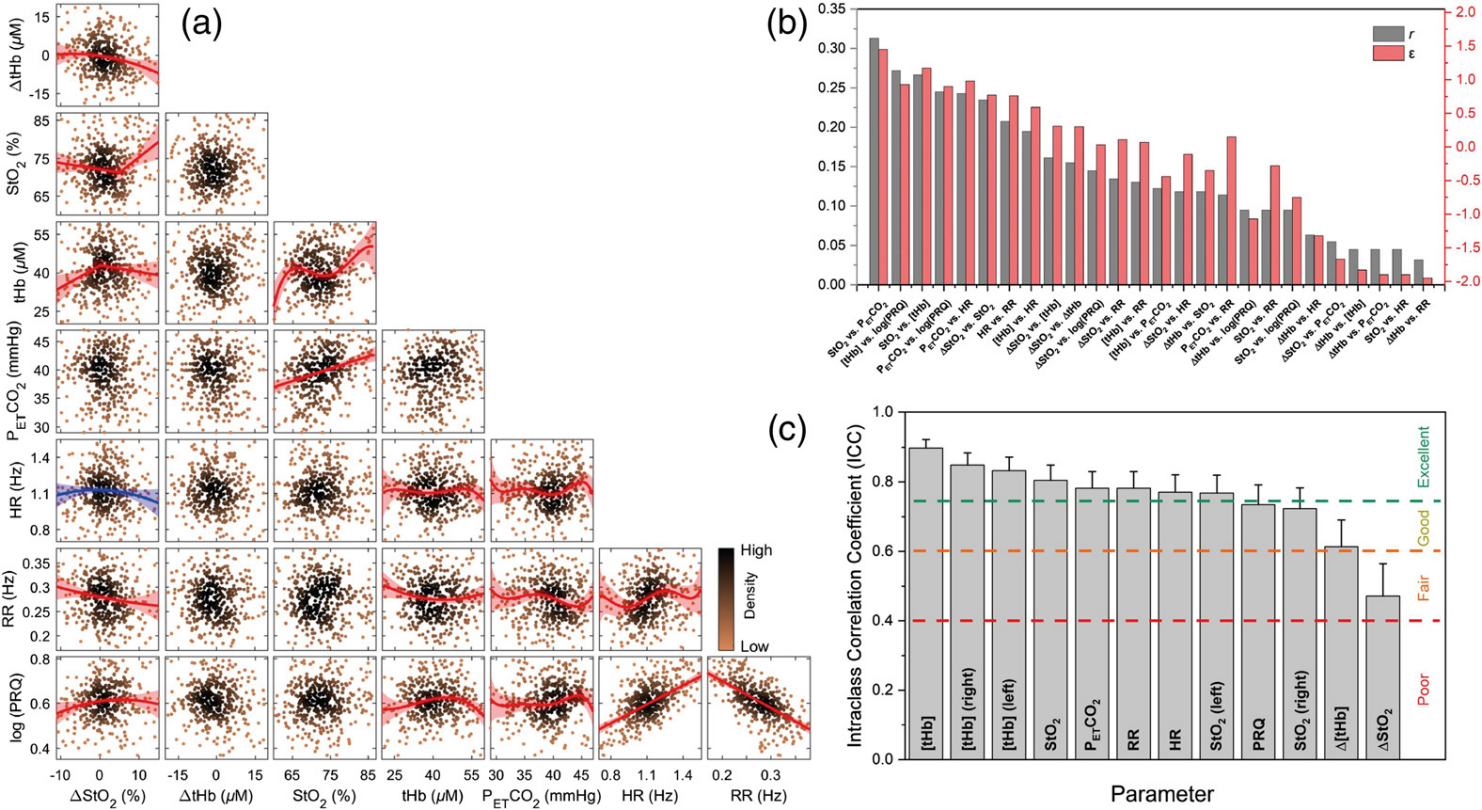
(a) Correlation matrix of bivariate scatter plots of $\Delta {\mathrm{StO}}_{2}$, ΔtHb, ${\mathrm{StO}}_{2}$, [tHb], $P_{\mathrm{ET}}{\mathrm{CO}}_{2}$, HR, RR, and log(PRQ). The best nonlinear fit is presented for pairs with a significant correlation (red: significant correlation proved by both FDR-corrected and uncorrected p values; blue: only uncorrected p value). The level of significance is calculated from goodness-of-fit results. The red and blue shaded areas show 95% of confidence intervals. Outliers are not displayed. (b) Bar chart of r- and ε values for each pair of $\Delta {\mathrm{StO}}_{2}$, ΔtHb, ${\mathrm{StO}}_{2}$, [tHb], $P_{\mathrm{ET}}{\mathrm{CO}}_{2}$, HR, RR, and log(PRQ) parameters (sorted in descending order of r-values). ε values <−0.5, near-zero (−0.5<ε<0.5), between 0.5 and 1, and >1 are indicative of no, inconclusive, moderate, and strong correlation, respectively. (c) Bar chart of ICC values for all cerebral and cardiorespiratory parameters. Error bars represent the 95% confidence interval. The reliability of the ICC is indicated.

A correlation matrix of $\Delta {\mathrm{StO}}_{2}$, ΔtHb, ${\mathrm{StO}}_{2}$, [tHb], $P_{\mathrm{ET}}{\mathrm{CO}}_{2}$, HR, RR, and log(PRQ) variables during the resting state is depicted in Fig. 3(a). In detail, Table S1 in the Supplementary Material shows curve fitted models, goodness-of-fit results, BSE parameter, and a significance level for each pair of variables. Statistically significant correlations were found between cerebral and cardiorespiratory parameters for six pairs of variables: $\Delta {\mathrm{StO}}_{2}$ versus RR ($p_{\mathrm{FDR}}=0.022$, p=0.010), $\Delta {\mathrm{StO}}_{2}$ versus log(PRQ) ($p_{\mathrm{FDR}}=0.024$, p=0.012), ${\mathrm{StO}}_{2}$ versus $P_{\mathrm{ET}}{\mathrm{CO}}_{2}$ ($p_{\mathrm{FDR}}<0.0001$, p<0.0001), [tHb] versus HR ($p_{\mathrm{FDR}}=0.001$, p<0.0004), [tHb] versus RR ($p_{\mathrm{FDR}}=0.029$, p=0.016), and [tHb] versus log(PRQ) ($p_{\mathrm{FDR}}<0.0001$, p<0.0001).

Correlations were also observed between the cerebral parameters: $\Delta {\mathrm{StO}}_{2}$ versus ΔtHb ($p_{\mathrm{FDR}}=0.008$, p=0.003), $\Delta {\mathrm{StO}}_{2}$ versus ${\mathrm{StO}}_{2}$ ($p_{\mathrm{FDR}}<0.0001$, p<0.0001), $\Delta {\mathrm{StO}}_{2}$ versus [tHb] ($p_{\mathrm{FDR}}=0.020$, p=0.008), and ${\mathrm{StO}}_{2}$ versus [tHb] ($p_{\mathrm{FDR}}<0.0001$, p<0.0001). A relatively strong correlation between cardiorespiratory parameters was also present: $P_{\mathrm{ET}}{\mathrm{CO}}_{2}$ versus HR ($p_{\mathrm{FDR}}<0.0001$, p<0.0001), $P_{\mathrm{ET}}{\mathrm{CO}}_{2}$ versus RR ($p_{\mathrm{FDR}}=0.039$, p=0.022), $P_{\mathrm{ET}}{\mathrm{CO}}_{2}$ versus log(PRQ) ($p_{\mathrm{FDR}}<0.0001$, p<0.0001), HR versus RR ($p_{\mathrm{FDR}}=0.001$, p<0.0005), HR versus log(PRQ) ($p_{\mathrm{FDR}}<0.0001$, p<0.0001), and RR versus log(PRQ) ($p_{\mathrm{FDR}}<0.0001$, p<0.0001). Since PRQ was calculated from HR and RR, we expected a linear correlation of HR versus log(PRQ), and RR versus log(PRQ). Additionally, Fig. 3(b) illustrates the coefficient of correlation “r” and BSE parameter “ε” for each pair. The order of correlation starting from strongest (r-value close to 1 and ε>0.5) is as follows: (1) ${\mathrm{StO}}_{2}$ versus $P_{\mathrm{ET}}{\mathrm{CO}}_{2}$, (2) ${\mathrm{StO}}_{2}$ versus [tHb], (3) $P_{\mathrm{ET}}{\mathrm{CO}}_{2}$ versus HR, (4) [tHb] versus log(PRQ), (5) $P_{\mathrm{ET}}{\mathrm{CO}}_{2}$ versus log(PRQ), (6) $\Delta {\mathrm{StO}}_{2}$ versus ${\mathrm{StO}}_{2}$, (7) HR versus RR, and (8) [tHb] versus HR.

### 3.4 Intrasubject Variability: ICC Values Indicate Good-to-Excellent Reliability of Most Parameters

Figure 3(c) presents the ICC values of all variables. The ICC of ${\mathrm{StO}}_{2}$, [tHb], ${\mathrm{StO}}_{2}$ (left), [tHb] (right), [tHb] (left), $P_{\mathrm{ET}}{\mathrm{CO}}_{2}$, HR, and RR indicates excellent reliability. The ICC of the PRQ, ${\mathrm{StO}}_{2}$ (right), and ΔtHb shows good and the ICC of $\Delta {\mathrm{StO}}_{2}$ represents moderate reliability.

### 3.5 Dependence of Cerebral Parameters on Sex and Seasonal Changes

**Fig. 4:**
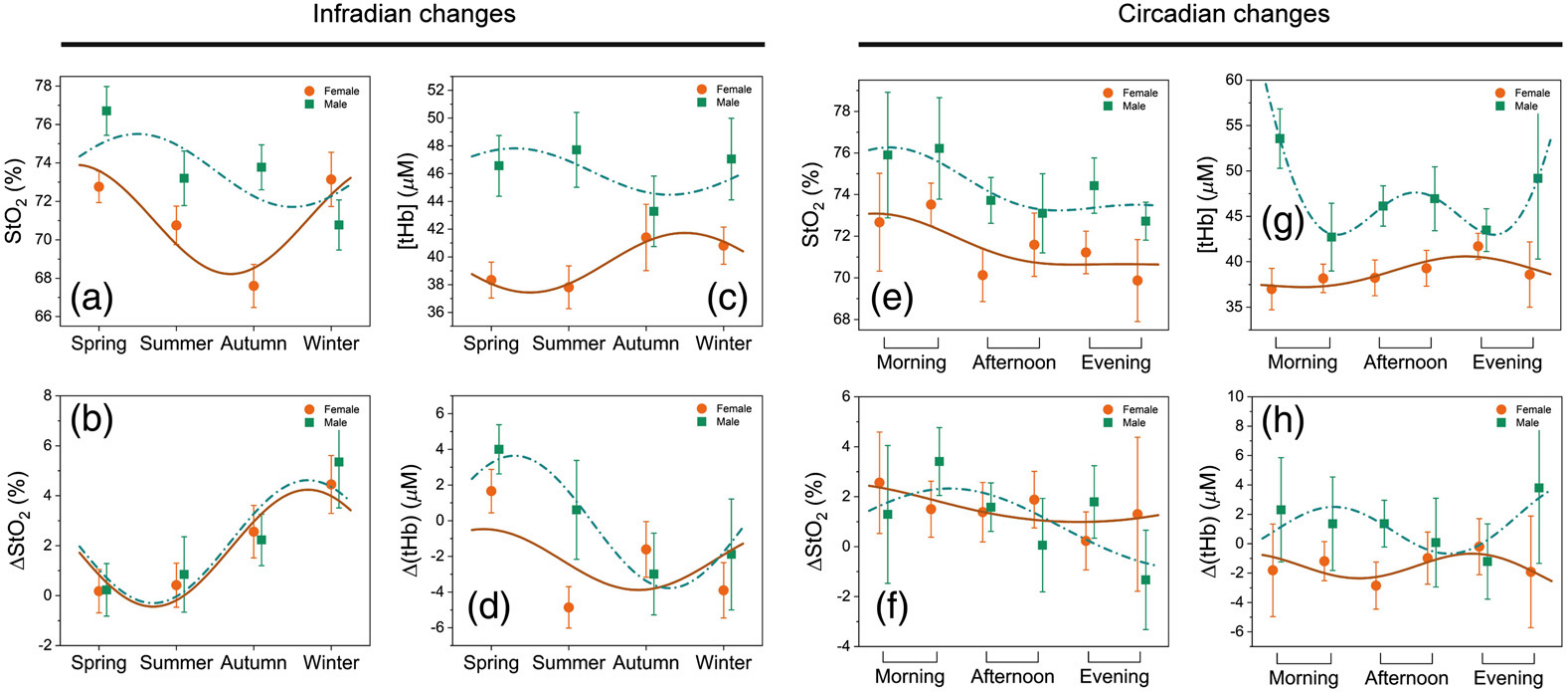
Changes in ${\mathrm{StO}}_{2}$, [tHb], $\Delta {\mathrm{StO}}_{2}$, and ΔtHb due to time of year [infradian changes (a)–(d)] and time of day [circadian changes (e)–(h); morning: 7:00–9:30 and 9:30–12:00; afternoon: 12:00–14:30 and 14:30–17:00; evening: 17:00–19:30 and 19:30–22:00] for females (orange) and males (green). Cosinor model and the sum of 2 cosines function are fitted to infradian and circadian changes, respectively (female, dark orange lines; male, dashed green lines). Error bars represent the 95% confidence interval.

The impact of sex and seasons (infradian changes) on cerebral parameters is depicted in Fig. 4. A significant difference due to sex (p<0.0001) was observed in both ${\mathrm{StO}}_{2}$ and [tHb]. In addition, Δ[tHb] was higher for males compared to females (p<0.001), but not for $\Delta {\mathrm{StO}}_{2}$ (p=0.635). We found that FCOA showed the same trend of seasonal changes for both female and male groups and was higher in autumn and winter compared to spring and summer (spring versus autumn, p=0.001; spring versus winter, p<0.001; summer versus autumn, p=0.024; summer versus winter, p<0.001 and autumn versus winter, p=0.011). Interestingly, a Cosinor model fitted to [tHb] data represents completely contrary patterns for male and female groups. Absolute [tHb] values of males were higher in spring and summer than that of in autumn and winter. Conversely, the [tHb] values of females were observed at higher levels in autumn and winter in comparison with spring and summer.

### 3.6 Dependence of Cerebral Parameters on Time of Day

The effect of time of day (circadian changes) on cerebral parameters is also shown in Fig. 4. A sum of 2 cosine functions was applied to fit the circadian rhythm of the data. The same trend of higher ${\mathrm{StO}}_{2}$ values in the morning was observed for males and females (p<0.001). Males demonstrated very high [tHb] values in the early morning and late evening (p<0.001), but the trend was almost opposite in females (p<0.05). The highest FCOA for [tHb] values were found at 10:00 and 14:00 for males and females, respectively. Regardless of sex, a highly significant difference was found between ${\mathrm{StO}}_{2}$ values of morning and afternoon (p<0.001). There was also a significant difference between ${\mathrm{StO}}_{2}$ values of morning and evening (p=0.011). No significant changes were found for [tHb] (morning versus afternoon: p=0.061; morning versus evening: p=0.17).

### 3.7 Dependence of Cerebral Parameters on Temperature

**Fig. 5:**
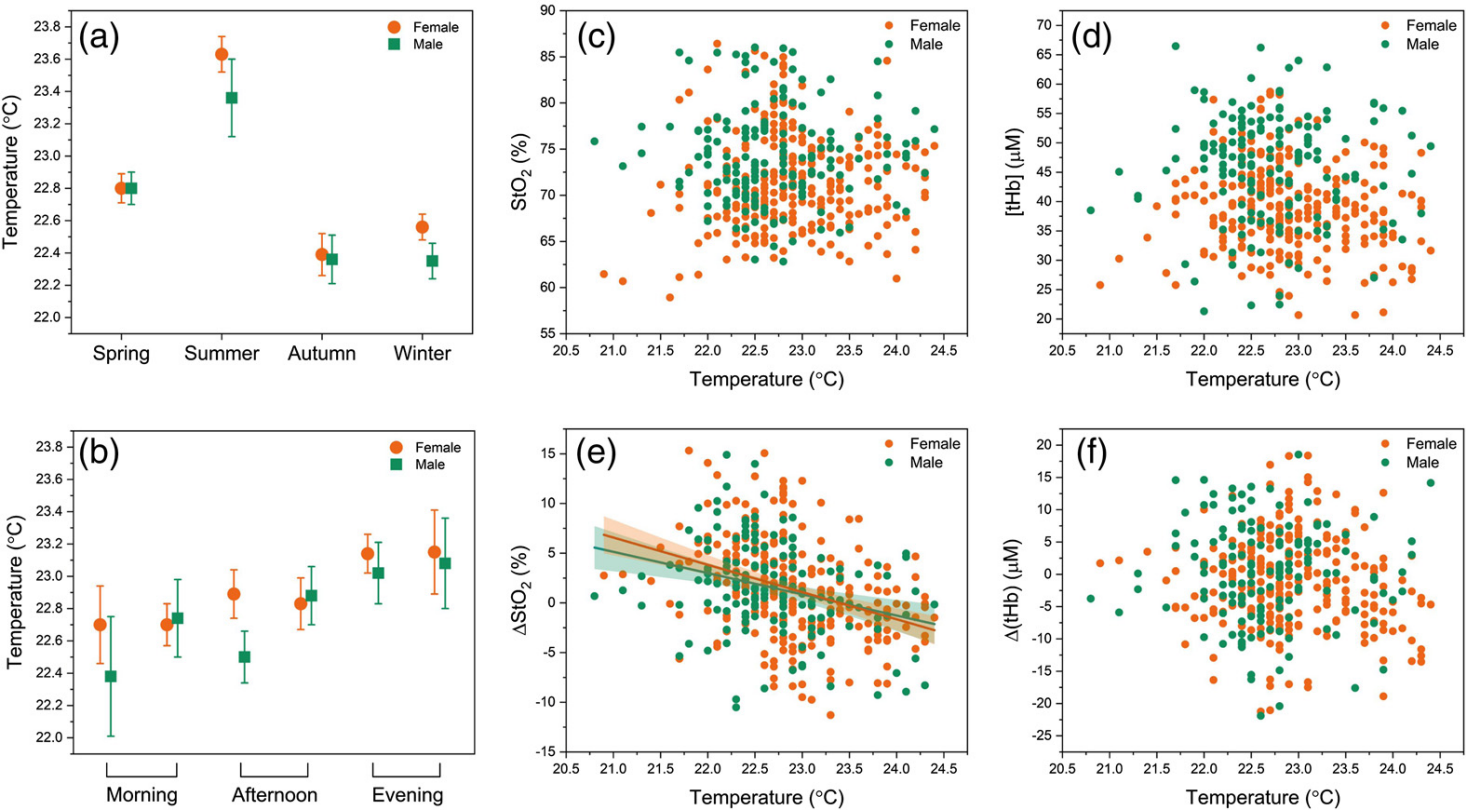
Changes in temperature with (a) time of year and (b) time of day. Dependence of (c) ${\mathrm{StO}}_{2}$, (d) [tHb], (e) $\Delta {\mathrm{StO}}_{2}$, and (f) ΔtHb on temperature. The lines represent a linear fit and the shaded areas and error bars show 95% confidence intervals. Outliers are not displayed.

Figure 5 shows the changes in room temperature with respect to season 5(a) and time of day 5(b), and the temperature dependency of cerebral parameters [5(c)–5(f)]. The mean room temperature was 22.8°C±0.6°C (range: 20.8°C to 24.8°C). Since the room was not air-conditioned, time of day and seasonal changes had an impact on the room temperature. As expected, the maximum room temperatures were recorded in summer and during the late evening. There was no significant linear correlation between room temperature and cerebral. The only exception was $\Delta {\mathrm{StO}}_{2}$, which decreased with increasing room temperature (females: r=−0.35, p<0.0001; males: r=−0.30, p<0.0002).

### 3.8 Dependence of Cerebral Parameters on Mood and Chronotype

**Fig. 6:**
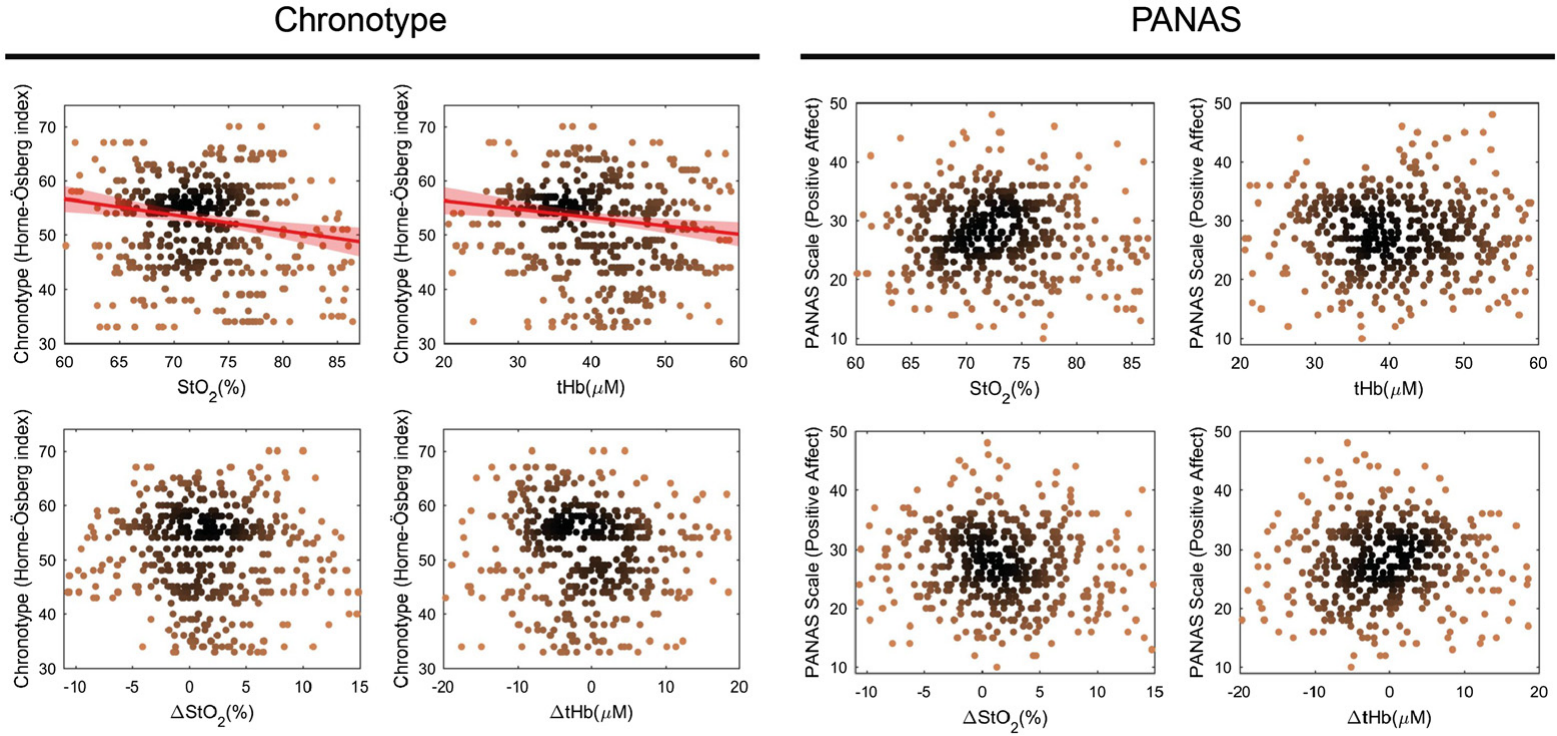
The scatter plots show the dependence of ${\mathrm{StO}}_{2}$, [tHb], $\Delta {\mathrm{StO}}_{2}$, and ΔtHb on chronotype (Horne-Östberg index) and mood (positive affect scale of PANAS). Correlations between the data are indicated by a linear fit, and the red shaded areas indicate 95% confidence intervals. Outliers are not displayed.

The mean valence, arousal, and dominance ratings during resting state assessed by SAM scales (ranging from 1 to 5) was 4.02±0.69, 2.92±0.91, and 3.24±0.77, respectively. The dependence of cerebral parameters on mood and chronotype is displayed in Fig. 6. No correlation was observed between the cerebral parameters and the positive affect scale of the PANAS questionnaire. The Horne and Östberg index was calculated for each subject to measure the chronotype. The highest numbers (score: 59 to 86) indicate morningness and the lowest numbers (score: 16 to 41) eveningness. Scores from 42 to 58 indicate neither morningness nor eveningness. We found a linear correlation between ${\mathrm{StO}}_{2}$ versus chronotype (r=−0.1, p=0.03), and [tHb] versus chronotype (r=−0.09, p=0.04).

## 4 Discussion

### 4.1 Absolute Values of Tissue Oxygenation and Hemoglobin Concentration

The normal range of ${\mathrm{StO}}_{2}$ and [tHb] of the brain was investigated for medical applications. These absolute values are approximately in accordance with the literature.^31^^,^^32^ The absolute ${\mathrm{StO}}_{2}$ values of the PFC are in good agreement with Choi et al. (R-PFC: 74.75%±5.83% versus. L-PFC: 75.63%±5.86%; N=30, age: 20 to 50 years, device: Imagent, ISS Inc.).^33^ However, our absolute [tHb] values at the PFC are lower in comparison with their findings (R-PFC: 79.68±12.15  μM, L-PFC: 76.93±14.98  μM). Our [tHb] values are similar to those of Vernieri et al. (left versus right frontal region: 46.2±11.9  μM versus 44.0±12.9  μM; 30 subjects, age: 63.9±8.2, device: Oximeter, ISS Inc.).^34^ Moreover, our ${\mathrm{StO}}_{2}$ values are a bit higher compared to our previous study [${\mathrm{StO}}_{2}$ (right) = 68.6% (IQR: 63.5% to 72.4%), ${\mathrm{StO}}_{2}$ (left) = 56.8% (IQR: 52.9% to 63.4%); 24 subjects, age: 22.0±6.4 years, device: OxiplexTS, ISS Inc.].^1^ The reasons for the difference between studies may be the age, physiological state of subjects, or methodology.

### 4.2 Absolute Values of Cardiorespiratory Parameters

Arterial partial pressure of carbon dioxide (${\mathrm{PaCO}}_{2}$) is one of the strongest parameters that affect CBF and [tHb].^19^ Therefore, ${\mathrm{PaCO}}_{2}$ has been included in functional brain studies to ensure a correct interpretation of the signals.^35^^–^^37^ We measured ${\mathrm{PaCO}}_{2}$ by the $P_{\mathrm{ET}}{\mathrm{CO}}_{2}$ method, which also provides continuous and noninvasive RR. Our $P_{\mathrm{ET}}{\mathrm{CO}}_{2}$ values were in agreement with the literature.^38^^–^^41^

Our findings showed that the mean value of HR was 68±11 BPM (range: 41 to 116 BPM, females: 69±11 BPM, males: 66±11 BPM; p=0.036). In 35,000 healthy subjects, a mean HR of 72 BPM (age: 20 and over, females: 74±0.2 BPM, males: 71±0.3 BPM; p<0.05) was determined, which is close to our results.^42^

The mean RR value measured in our study was 16.5±2.9 BrPM, which is within typical RR for adults (range: 6.9 to 27.1 BrPM, females: 16.8±2.8 BPM, males: 16.1±3.1 BPM; p=0.015).

The PRQ is a parameter to attain the overall current state of human physiology.^43^ PRQ represents the state of the ANS and is a measure of cardiorespiratory coordination. PRQ is time- and sex-dependent, and changes during human development, physical activity, and body posture with specific patterns during sleep.^43^ The resting state PRQ distribution has a peak at ∼4.^44^^–^^46^ We also found a mean resting state PRQ of ∼4 (4.2±0.9, ranging from 2.1 to 9.0).

### 4.3 Relationships with Systemic Physiology

Although systemic physiological activity affects the absolute values of ${\mathrm{StO}}_{2}$ and [tHb], FCOA was not influenced. The reason is that both R-PFC and L-PFC are affected in the same way by systemic physiology.^1^^,^^21^ Although we generally confirmed this finding, we found nonlinear correlations between $\Delta {\mathrm{StO}}_{2}$ versus RR (p=0.022) and $\Delta {\mathrm{StO}}_{2}$ versus PRQ (p=0.024).

As expected, we found a highly significant ($R^{2}=0.10$, p<0.0001) positive linear correlation between ${\mathrm{StO}}_{2}$ and $P_{\mathrm{ET}}{\mathrm{CO}}_{2}$. Our finding is in agreement with the study carried out by Miller and Mitra.^47^ and with the physiologically well-known ${\mathrm{CO}}_{2}$-response, i.e., a decrease in $P_{\mathrm{ET}}{\mathrm{CO}}_{2}$ (hypocapnia) reduces the CBF by cerebral vasoconstriction.^48^ This reduced oxygen supply leads to a lower ${\mathrm{StO}}_{2}$.^35^^,^^36^ Hence, $P_{\mathrm{ET}}{\mathrm{CO}}_{2}$ is positively correlated with ${\mathrm{StO}}_{2}$

### 4.4 Frontal Cortex Oxygenation Asymmetry

In EEG studies, activity in left frontal regions is mostly associated with appetitive motivation and approach-related affect such as hope, happiness, and joy (positive affect). Conversely, the right frontal regions are related to vigilant attention and behavioral inhibition that regularly occurs during certain withdrawal-related affect such as depression and nervousness (negative affect).^11^^,^^12^^,^^49^^–^^51^ In general, the right frontal cortex reflects motivational systems of approach and avoidance, whereas the left frontal cortex inhibits the amygdala and downregulates negative affect.^52^^,^^53^ Higher right frontal activity is attributed to greater negative affect (e.g., film-induced fear and disgust), whereas positive affect (e.g., film-induced happiness) elicits a higher left frontal activity.^54^^,^^55^

We hypothesized that the FCOA reveals asymmetry of the PFC neuronal activity at rest. The ${\mathrm{StO}}_{2}$ was higher at the R-PFC than the L-PFC, indicating that the R-PFC is more activated than the L-PFC and this indicates a higher inhibitory activity or withdrawal motivation. This is reasonable considering that the subjects were in the resting state. Such rightward lateralization has also been found in the literature.^56^^,^^57^ Although our findings are in line with several studies indicating that the right cortices have a stronger response compared to the left ones, some studies have reported no hemispheric differences or even leftward regional lateralization.^33^^,^^58^ Liu et al.^6^ demonstrated that right or left regional laterality could be observed across different brain systems depending on multiple genetic or environmental mechanisms.

#### 4.4.1 FCOA as an indicator of human health

The R-PFC plays a vital role in the brain’s response to stress because this area is a primary part of both the emotion and vigilance networks. Neurons that are either the target or the releasing site of an array of stress mediators (neurotransmitter and hormone) have been recognized in this area.^59^ Thus FCOA is associated with specific emotional responses to mental stress and personality traits (state influence versus trait influence).^14^^,^^52^^,^^60^ High left frontal brain activity is more psychologically and physically healthy than relatively less left frontal brain activity.^53^^,^^61^ Individuals with higher L-PFC activity have lower concentrations of the stress hormone cortisol and the corticotrophin-releasing hormone, higher activity of natural killer cells, and higher antibody concentration in response to influenza vaccines.^53^^,^^62^ It was also demonstrated that subjects with higher L-PFC activity, recover more quickly from a negative occurrence with higher levels of psychological well-being.^53^^,^^63^ Conversely, dominant R-PFC activity is associated with increased activation of the hypothalamic–pituitary–adrenal axis^59^^,^^64^^–^^66^ and higher secretion of corticotrophin-releasing hormone and adrenal steroid hormones (e.g., glucocorticoids and adrenal androgens).^66^^–^^69^ Higher R-PFC activity may occur during stressful situations, such as a test or job interview.^70^ An EEG study showed that FEA was shifted from the left during an easy examination session to the right during a stressful examination session.^71^ A higher change in FEA from the easy to the stressful session was associated with more adverse health conditions. Further research suggested that subjects with more R-PFC activity compared to L-PFC are sensitive to mental stress and prone to exhibit various stress-induced somatic disorders.^56^^,^^64^ Moreover, it was found that higher levels of R-PFC activation predict a reduced immune response in humans.^59^^,^^62^ The more an individual’s FEA is changed during periods of stress, the more negative health consequences are likely to be experienced.^49^

We observed no correlation between cerebral parameters; in particular, FCOA and the positive affect scale of the PANAS questionnaire.

Depression is associated with an under-activation of the approach system and/or over-activation of the withdrawal system.^72^ Research provides support for an association between FEA and depression^12^^,^^73^^,^^74^ and may predict the emotional state in depression disorders.^72^ Although there is a small number of studies linking FEA with psychopathology, the research suggests FEA may be a promising marker of depression vulnerability.^49^ Decreased relative left-frontal activity during resting state was attributed to increased vulnerability to depression.^75^ In adolescent boys without a history of depression, right-sided frontal activation predicted depressive symptoms 1 year later.^76^ Many studies indicate that FEA is a valid marker for depression vulnerability. Regardless of whether anxiety was used as a covariate or not, frontal alpha asymmetry indicative of relatively higher right frontal activity predicts depression, whereas the opposite is not true.^77^

Thus the measurement of such an FEA and FCOA may have considerable clinical value.

#### 4.4.2 Anatomic, physiologic, and genetic influences

The corpus callosum provides a neuroanatomical correlation in the asymmetry of the frontal cortices.^78^ Negative affect generally leads to activation in the R-PFC, amygdala, inferior frontal gyrus, and insula, whereas the L-PFC may play a role in the downregulation of amygdala and is associated with reward-related cortical regions.^53^^,^^65^^,^^79^ The neural correlates of vigilance and sustained attention are primarily localized in the right prefrontal and parietal lobe and the thalamus.^80^ The link between left frontal and left amygdala activity is crucial for emotional regulation.^73^

Genetic models have been proposed to account for cerebral dominance, and anatomical asymmetries are likely influenced by genetic factors. However, no gene or pathway has yet been identified as a determinant of lateralization, although there are a number of candidates including LMO4, STMN4, BAI1, and IGFBP5, which were highly expressed in the right regions.^3^^,^^81^^,^^82^

#### 4.4.3 FEA as a promising marker of subject characteristics and emotions

It was demonstrated that asymmetry in PFC neuronal activity during the resting state, measured with EEG, predicts the emotional state.^72^

Table 1 shows a summary of the emotions and characteristics of individuals with FEA.

**Table 1:** A summary of subject characteristics and emotions with left and right dominant activity

Left dominant activity	Right dominant activity	References
Anxious apprehension (e.g., worry)	Anxious arousal (e.g., panic)	72, 73, and 83–86
Maniacs	Phobias (social phobics)	50, 83, 87 and 88
Extrovert	Introvert (neuroticism)	89 and 90
Promotion (a need for growth and advancement)	Prevention (a need for safety and security)	91 and 92
Anger, joy, and jealousy	Disgust and depression	12 and 93–97
Hostility to social rejection	Isolation to social rejection	60 and 98
Higher socioeconomic status	Lower socioeconomic status	99
—	Defensiveness	100 and 101
—	Hopelessness	75 and 102
—	Less risk-taking	103
—	Ostracism	104
—	Obsessive-compulsive disorder	105
—	More concerned with making mistakes and punishment	106

#### 4.4.4 Environment and certain situational variable

Experimental, environmental, and situational factors that influence approach or withdrawal motivation may affect FCOA. These variables include body posture, experimental conditions, trait variables, and timing. Thus the seasons and time of day play essential roles in FCOA scores. We found higher R-PFC activity (higher ${\mathrm{StO}}_{2}$) associated with more depression in autumn and winter compared to spring and summer. These findings are in line with a previous study.^107^ Indeed, it is known that the population experiences a worsening of their mood and stronger depressive symptoms (seasonal affective disorder) in winter.^108^^–^^110^ This is also visible in the highly significant seasonal variation in cortisol levels in winter and autumn compared to spring and summer.^111^ Thus it is reasonable that we found a significant influence of the seasons on FCOA.

We also found a significant dependence of the ${\mathrm{StO}}_{2}$ on the time of day (Fig. 4). Higher ${\mathrm{StO}}_{2}$ values were observed in the morning compared to the afternoon and evening. Since [tHb] was not significantly changed during the day, such an increase in ${\mathrm{StO}}_{2}$ may imply that more oxygenated blood was present in the brain tissue during the morning hours compared to the evening and afternoon. We can interpret this increase as reduced oxygen consumption and, thus, energy metabolism in the morning, which would be in agreement with the synaptic homeostasis hypothesis.^112^ This increase could also be linked to circadian effects, e.g., cortisol rhythm exerting its wake-promoting effect in the morning hours.^113^^,^^114^ Moreover, the present findings indicated that the time of day has no significant influence on FCOA, which is in line with the literature.^115^

It was also shown in this study that FCOA in ${\mathrm{StO}}_{2}$ decreased with increasing room temperature. In other words, we found that the lower the room temperature, the higher the R-PFC activation. Lower room temperature increased whole-body cooling sensation and reduced thermal comfort, especially after prolonged exposure.^116^ Our findings are in line with the approach-motivational model, which links higher R-PFC activation to greater withdrawal-related affect (negative affect) such as uncomfortableness, nervousness, and depression. It is known that increasing the length of daylight and temperature results in a decrease in the depression score,^110^^,^^117^ which indirectly confirms our findings in terms of the effects of both seasons and temperature on FCOA.

## 5 Conclusion

We found highly significant (p<0.0001) FCOA, which was correlated to room temperature, RR, and PRQ but was not affected by mood or chronotype of the subject. This higher right PFC activity may be due to the more prominent inhibition activity during the resting state.

The absolute values of ${\mathrm{StO}}_{2}$ and [tHb] were influenced by systemic physiological activity, such as $P_{\mathrm{ET}}{\mathrm{CO}}_{2}$, HR, RR, and PRQ, and gender.

FCOA and ${\mathrm{StO}}_{2}$ were dependent on season and time of day, respectively. FCOA was higher in autumn/winter compared to spring/summer, whereas ${\mathrm{StO}}_{2}$ was higher in the morning than in the afternoon/evening.

These relevant findings were only achievable using FD-fNIRS instrumentation that enabled the measurement of absolute values while using a SPA-fNIRS approach.

Our study demonstrates that FCOA is real, while providing unique insights to understand this remarkable phenomenon.
