# Supplementary material for: Frontal cerebral oxygenation asymmetry: intersubject variability and dependence on systemic physiology, season, and time of day
Source: Neurophotonics. 2020 Jun 23;7(2):025006. doi: 10.1117/1.NPh.7.2.025006 (PMC7310879; doi:10.1117/1.NPh.7.2.025006)
Supplement: Supplementary file 1 [file NPh_007_025006_SD001.docx]

Table S1: Curve fitted models, goodness-of-fit results, bootstrapped evidence parameter, and a significance level for each pair of parameters

| **Para- meter 1** | **Para- meter 2** | **Model** | **Model parameters (95% CI)** | **R^2^** | **RMSE** | ***P* value** | **BSE, ε** |
| --- | --- | --- | --- | --- | --- | --- | --- |
| ΔStO_2_ | ΔtHb | $f\left( x \right)=p_{1}x^{2}+p_{2}x+p_{3}$ | \| $p_{1}=-0.015 \left( -0.034, 0.003 \right)$ \| \| --- \| \| $p_{2}=-0.220 (-0.368, -0.072)$  $p_{3}=-0.391 (-1.209, 0.426)$ \| | 0.024 | 7.123 | 0.008** | 0.30  (inconclusive) |
| ΔStO_2_ | StO_2_ | $f\left( x \right)=\left\{ \begin{aligned} p_{1}x+ p_{2} (x<x_{0}) \\ p_{3}x+ p_{4} (x>x_{0}) \end{aligned} \right.$ | \| $p_{1}=-0.173 (-0.328, -0.017)$ \| \| --- \| \| $p_{2}=72.114 (71.548, 72.679)$  $x_{0}=4.932 (2.884, 6.980)$ \| \| $p_{3}=0.764 (0.372, 1.157)$  $p_{4}=67.488 \left( 64.133, 70.842 \right)$ \| | 0.055 | 5.311 | 4.9e-05*** | 0.77  (moderate H_1_) |
| ΔStO_2_ | [tHb] | $f\left( x \right)=\left\{ \begin{aligned} p_{1}x+ p_{2} (x<x_{0}) \\ p_{3}x+ p_{4} (x>x_{0}) \end{aligned} \right.$ | \| $p_{1}=0.621 (0.190, 1.051)$ \| \| --- \| \| $p_{2}=42.650 (40.791, 44.509)$  $x_{0}=0.108 (-2.860, 3.077)$ \| \| $p_{3}=-0.246 (-0.559, 0.066)$  $p_{4}=42.744 (40.970, 44.518)$ \| | 0.026 | 8.632 | 0.020* | 0.31  (inconclusive) |
| ΔStO_2_ | P_ET_CO_2_ | $f\left( x \right)=p_{1}x+p_{2}$ | \| $p_{1}=-0.047 (-0.126, 0.030)$ \| \| --- \| \| $p_{2}=39.38 (38.98, 39.78)$ \| | 0.003 | 4.056 | 0.266 | -1.67  (overwhelming H_0_) |
| ΔStO_2_ | HR | $f\left( x \right)=p_{1}x^{2}+p_{2}x+p_{3}$ | \| $p_{1}=-0.000 (-0.001, 0.000)$ \| \| --- \| \| $p_{2}=-0.001 (-0.004, 0.002)$  $p_{3}=1.128 (1.108, 1.147)$ \| | 0.014 | 0.167 | 0.052 | -0.11  (inconclusive) |
| ΔStO_2_ | RR | $f\left( x \right)=p_{1}x^{2}+p_{2}x+p_{3}$ | \| $p_{1}=-0.000 (-0.000, 0.000)$ \| \| --- \| \| $p_{2}=-0.001 (-0.002, -0.000)$  $p_{3}=0.279 (0.274, 0.284)$ \| | 0.018 | 0.044 | 0.022* | 0.11  (inconclusive) |
| ΔStO_2_ | log(PRQ) | $f\left( x \right)=p_{1}x^{2}+p_{2}x+p_{3}$ | \| $p_{1}=-0.000 (-0.000,-0.000)$ \| \| --- \| \| $p_{2}=0.002 (0.001, 0.004)$  $p_{3}=0.609 (0.599, 0.619)$ \| | 0.021 | 0.084 | 0.024* | 0.03  (inconclusive) |
| ΔtHb | StO_2_ | $f\left( x \right)=p_{1}x^{3}+p_{2}x^{2}+p_{3}x+p_{4}$ | \| $p_{1}=-0.000 (-0.001, 0.000)$ \| \| --- \| \| $p_{2}=0.006 (-0.001, 0.013)$  $p_{3}=0.111 (-0.005, 0.227)$  $p_{4}=72.03 (71.4, 72.65)$ \| | 0.014 | 5.326 | 0.087 | -0.35  (inconclusive) |
| ΔtHb | [tHb] | $f\left( x \right)=p_{1}x^{2}+p_{2}x+p_{3}$ | \| $p_{1}=0.003 (-0.007, 0.013)$ \| \| --- \| \| $p_{2}=0.050 (-0.061, 0.162)$  $p_{3}=40.98 (39.98, 41.98)$ \| | 0.002 | 8.79 | 0.598 | -1.83  (overwhelming H_0_) |
| ΔtHb | P_ET_CO_2_ | $f\left( x \right)=p_{1}x+p_{2}$ | \| $p_{1}=-0.023 (-0.075, 0.029)$ \| \| --- \| \| $p_{2}=39.31 (38.92, 39.7)$ \| | 0.002 | 4.073 | 0.414 | -1.90  (overwhelming H_0_) |
| ΔtHb | HR | $f\left( x \right)=p_{1}x+p_{2}$ | \| $p_{1}= -0.001 (-0.003, 0.0004)$ \| \| --- \| \| $p_{2}= 1.115 (1.099, 1.131)$ \| | 0.004 | 0.165 | 0.176 | -1.32  (Compelling H_0_) |
| ΔtHb | RR | $f\left( x \right)=p_{1}x+p_{2}$ | \| $p_{1}= -0.0002 (-0.000, 0.000)$ \| \| --- \| \| $p_{2}= 0.275 (0.271, 0.279)$ \| | 0.001 | 0.044 | 0.592 | -1.95  (overwhelming H_0_) |
| ΔtHb | log(PRQ) | $f\left( x \right)=p_{1}x^{2}+p_{2}x+p_{3}$ | \| $p_{1}=-0.0001(-0.000, 0.000)$ \| \| --- \| \| $p_{2}=-0.001 (-0.002, 0.000)$  $p_{3}=0.614 (0.604, 0.624)$ \| | 0.009 | 0.083 | 0.160 | -1.07  (Compelling H_0_) |
| StO_2_ | [tHb] | $f\left( x \right)=p_{1}x^{4}+p_{2}x^{3}+p_{3}x^{2}+p_{4}x+p_{5}$ | \| $p_{1}=-0.001 (-0.001, -0.000)$ \| \| --- \| \| $p_{2}=0.214 (0.102, 0.325)$  $p_{3}=-23.97 (-36.09, -11.84)$  $p_{4}=1187 (603.7, 1771)$  $p_{5}=-2.19e+04$ \| | 0.071 | 8.51 | 2.3e-07*** | 1.17  (Compelling H_1_) |
| StO_2_ | P_ET_CO_2_ | $f\left( x \right)=p_{1}x+p_{2}$ | \| $p_{1}= 0.217 (0.151, 0.283)$ \| \| --- \| \| $p_{2}= 23.85 (19.04, 28.67)$ \| | 0.098 | 3.86 | 3.1e-11*** | 1.45  (Compelling H_1_) |
| StO_2_ | HR | $f\left( x \right)=p_{1}x+p_{2}$ | \| $p_{1}= 0.001 (-0.001, 0.004)$ \| \| --- \| \| $p_{2}= 1.018 (0.804, 1.232)$ \| | 0.002 | 0.168 | 0.406 | -1.90  (overwhelming H_0_) |
| StO_2_ | RR | $f\left( x \right)=p_{1}x^{3}+p_{2}x^{2}+p_{3}x+p_{4}$ | \| $p_{1}=-0.000 (-0.000, -0.000)$ \| \| --- \| \| $p_{2}=0.005 (0.002, 0.008)$  $p_{3}=-0.392 (-0.614, -0.171)$  $p_{4}=9.636 (4.277, 14.99)$ \| | 0.009 | 0.044 | 0.087 | -0.28  (inconclusive) |
| StO_2_ | log(PRQ) | $f\left( x \right)=p_{1}x^{3}+p_{2}x^{2}+p_{3}x+p_{4}$ | \| $p_{1}=-0.000 (-0.000, 0.000)$ \| \| --- \| \| $p_{2}=-0.006 (-0.012, 0.001)$  $p_{3}=0.410 (-0.034, 0.853)$  $p_{4}=-9.505 (-20.23, 1.225)$ \| | 0.009 | 0.085 | 0.189 | -0.75  (moderate H_0_) |
| [tHb] | P_ET_CO_2_ | $f\left( x \right)=p_{1}x^{3}+p_{2}x^{2}+p_{3}x+p_{4}$ | \| $p_{1}=-0.0002 (-0.0005, 0.000)$ \| \| --- \| \| $p_{2}=0.033 (-0.004, 0.071)$  $p_{3}=-1.347 (-2.902, 0.208)$  $p_{4}=56.12 (35.39, 76.86)$ \| | 0.015 | 4.063 | 0.122 | -0.44  (inconclusive) |
| [tHb] | HR | $f\left( x \right)=p_{1}x^{4}+p_{2}x^{3}+p_{3}x^{2}+p_{4}x+p_{5}$ | \| $p_{1}=-0.0002 (-0.0005, 0.000)$ \| \| --- \| \| $p_{2}=0.033 (-0.004, 0.071)$  $p_{3}=-1.347 (-2.902, 0.208)$  $p_{4}=56.12 (35.39, 76.86)$  $p_{5}=-0.755 (-3.408, 1.898)$ \| | 0.038 | 0.162 | 0.001** | 0.59  (moderate H_1_) |
| [tHb] | RR | $f\left( x \right)=p_{1}x^{2}+p_{2}x+p_{3}$ | \| $p_{1}=0.000 (0.000, 0.000)$ \| \| --- \| \| $p_{2}=-0.004 (-0.008, -0.001)$  $p_{3}=0.381 (0.31, 0.452)$ \| | 0.017 | 0.043 | 0.029* | 0.07  (inconclusive) |
| [tHb] | log(PRQ) | $f\left( x \right)=p_{1}x^{3}+p_{2}x^{2}+p_{3}x+p_{4}$ | \| $p_{1}=-0.000 (-0.000, -0.000)$ \| \| --- \| \| $p_{2}=0.001 (0.000, 0.002)$  $p_{3}=-0.023 (-0.054, 0.008)$  $p_{4}=0.787 (0.370, 1.204)$ \| | 0.074 | 0.081 | 2.6e-06*** | 0.93  (moderate H_1_) |
| P_ET_CO_2_ | HR | $f\left( x \right)=p_{1}x^{5}+p_{2}x^{4}+p_{3}x^{3}+p_{4}x^{2}+p_{5}x+p_{6}$ | \| $p_{1}=-0.000 (-0.000,-0.000)$ \| \| --- \| \| $p_{2}=0.0018 (0.0009, 0.0028)$  $p_{3}=-0.137 (-0.208, -0.065)$  $p_{4}=5.088 (2.356, 7.819)$  $p_{5}=-94.11 \left( -146, 42.27 \right)$  $p_{6}=693.3 (302.2, 1084)$ \| | 0.059 | 0.160 | 4.9e-05*** | 0.98  (moderate H_1_) |
| P_ET_CO_2_ | RR | $f\left( x \right)=p_{1}x^{4}+p_{2}x^{3}+p_{3}x^{2}+p_{4}x+p_{5}$ | \| $p_{1}=0.000 (0.000, 0.000)$ \| \| --- \| \| $p_{2}=-0.0017 (-0.0027, -0.0006)$  $p_{3}=0.093 (0.032, 0.154)$  $p_{4}=-2.299 (-3.84, -0.758)$  $p_{5}=21.41 (6.845, 35.98)$ \| | 0.013 | 0.043 | 0.039* | 0.15  (inconclusive) |
| P_ET_CO_2_ | log(PRQ) | $f\left( x \right)=p_{1}x^{5}+p_{2}x^{4}+p_{3}x^{3}+p_{4}x^{2}+p_{5}x+p_{6}$ | \| $p_{1}=-0.000 (-0.000,-0.000)$ \| \| --- \| \| $p_{2}=0.0006 (0.0001, 0.0011)$  $p_{3}=-0.045 (-0.082, -0.008)$  $p_{4}=1.666 (0.2457, 3.086)$  $p_{5}=-30.53 \left( -57.48, -3.569 \right)$  $p_{6}=223.2 (19.84, 426.6)$ \| | 0.060 | 0.082 | 2.2e-05*** | 0.90  (moderate H_1_) |
| HR | RR | $f\left( x \right)=p_{1}x^{5}+p_{2}x^{4}+p_{3}x^{3}+p_{4}x^{2}+p_{5}x+p_{6}$ | \| $p_{1}=7.775 (-0.3543,15.91)$ \| \| --- \| \| $p_{2}=-42.26 (-88.32, 3.789)$  $p_{3}=89.63 (-13.34, 192.6)$  $p_{4}=-92.49 (-206, 21.02)$  $p_{5}=46.37 \left( -15.28, 108 \right)$  $p_{6}=-8.759 (-21.95, 4.428)$ \| | 0.043 | 0.043 | 0.001** | 0.76  (moderate H_1_) |
| HR | log(PRQ) | $f\left( x \right)=p_{1}x+p_{2}$ | \| $p_{1}= 0.283 (0.245, 0.320)$ \| \| --- \| \| $p_{2}= 0.284 (0.242, 0.326)$ \| | 0.358 | 0.067 | 5.0e-46*** | 2.19  (overwhelming H_1_) |
| RR | log(PRQ) | $f\left( x \right)=p_{1}x+p_{2}$ | \| $p_{1}= -1.208 (-1.347, -1.069)$ \| \| --- \| \| $p_{2}= 0.942 (0.903, 0.981)$ \| | 0.42 | 0.064 | 4.9e-55*** | 2.52  (overwhelming H_1_) |
